# Selective bladder preservation for muscle-invasive transitional cell carcinoma of the urinary bladder

**DOI:** 10.1038/sj.bjc.6601580

**Published:** 2004-02-03

**Authors:** M D Michaelson, W U Shipley, N M Heney, A L Zietman, D S Kaufman

**Affiliations:** 1 Departments of Hematology/Oncology, Radiation Oncology and Urology, Massachusetts General Hospital Cancer Center, Harvard Medical School, Boston, MA, USA

**Keywords:** bladder cancer, radiation therapy, chemotherapy, radical cystectomy, cisplatin

## Abstract

Invasive transitional cell carcinoma (TCC) of the urinary bladder is traditionally treated with radical cystectomy. This approach results in great morbidity and lifestyle changes, and approximately half of the patients treated in this way will experience recurrent TCC despite surgery. An alternative approach using selective bladder-preservation techniques incorporates transurethral resection of bladder tumours, radiation therapy, and chemotherapy. Over the past 20 years, international experience has demonstrated that this approach is feasible, safe, and well tolerated. Furthermore, the long-term outcomes of overall survival and disease-free survival compare favourably with the outcomes from radical cystectomy. The most important predictor of response is stage, with significantly higher long-term survival in patients with T2 disease. Another important positive predictor of complete response to therapy is the ability of the urologic oncologist to remove all visible tumour through a transurethral approach prior to initiation of radiation therapy. A negative predictive factor is the presence of hydronephrosis, and age and gender do not affect disease-free survival. The majority of patients who enjoy long-term survival do so with an intact native bladder. Quality of life studies have demonstrated that the retained bladder functions well in nearly all of these patients. Selective bladder preservation will not entirely take the place of radical cystectomy, but should be offered as an important alternative to patients newly diagnosed with muscle-invasive TCC.

Radical cystectomy has been the standard treatment for muscle invasive transitional cell carcinoma (TCC) of the bladder. Radical surgery results, historically, in a long-term survival rate of only 40–60% (Pagano *et al*, 1991; Waehre *et al*, 1993). More recent analysis of radical cystectomy at a single institution demonstrated a disease-specific survival of 67% with a median follow-up of 65 months, and long-term overall survival of 45% (Dalbagni *et al*, 2001). Another single-institution series stratified patients by pathologic stage, and found a recurrence-free survival of 89% in pathologic T2, node-negative tumours, 50% in T4 node-negative tumours, and 35% in patients with node-positive tumours (Stein *et al*, 2001). The negative impact that urinary diversion has on lifestyle is part of the impetus for a search for alternatives.

Cystoscopic tumour resection alone or radiation therapy alone provide inferior outcomes to radical cystectomy, with only 20–40% local control rates in muscle invasive TCC. However, an accumulation of international experience over the past two decades has established that trimodality bladder preservation treatment is a legitimate alternative in selected cases of patients with muscle-invasive TCC. The algorithm for this approach is initial cystoscopic resection of as much bladder tumour as is safely possible, followed by a combination of bladder irradiation with concurrent radiosensitising chemotherapy, followed by adjuvant chemotherapy. Active cystoscopic surveillance of the bladder is maintained throughout the treatment period, and radical cystectomy is advised in the event of persistent invasive disease. Although randomised studies comparing this approach to surgery have not been performed, survival outcomes with selective bladder preservation are comparable to outcomes from radical cystectomy in comparable patients. This review will focus on studies of selective bladder-preserving treatment of muscle-invasive TCC, as well as on quality of life measures in patients who have undergone this treatment.

## INTERNATIONAL EXPERIENCE WITH SELECTIVE BLADDER PRESERVATION

Selective bladder preservation for muscle-invasive TCC in North America has occurred primarily under the auspices of the Radiation Therapy Oncology Group (RTOG) and the National Bladder Cancer Group. A series of six consecutive RTOG clinical trials has been completed to date. The initial study RTOG 85-12 treated 42 patients with daily radiation therapy and concurrent cisplatin, and reported greater than 50% 5-year survival (Tester *et al*, 1993). The approach was feasible, well tolerated by patients, and resulted in 42% long-term survival with an intact bladder. Subsequent studies conducted by the RTOG have explored numerous additional questions, including the addition of other chemotherapy agents to cisplatin, including 5-fluorouracil (5FU) and paclitaxel, the feasibility of outpatient treatment with this approach, the utility of neoadjuvant or adjuvant chemotherapy, the use of hyperfractionated radiation therapy, and others. These studies will be further described below.

Concurrently, a number of European groups engaged in pioneering efforts in bladder-preserving approaches to treatment. The University of Paris treated 54 patients with transurethral surgery, followed by concomitant radiation and chemotherapy (Housset *et al*, 1993). In this study, 5FU was added to cisplatin, and twice daily radiation was employed. Treatment was well tolerated, and disease-free survival was 62% at 3 years.

The University of Erlangen reported on their 10-year experience of 79 patients treated with transurethral bladder resection followed by daily radiation with concurrent cisplatin (Dunst *et al*, 1994). Results were similar to those of the RTOG and the University of Paris, with 52% 5-year overall survival and 41% survival with intact bladder. Their experience was expanded in a subsequent report in which 162 patients were treated with either cisplatin or carboplatin (Sauer *et al*, 1998). The overall 5-year survival was 55%, and 44% of total patients survived long term with an intact bladder. Patients treated with carboplatin did not have as favourable an outcome as those treated with cisplatin, although the numbers in each group were too small for meaningful statistical comparison. A subsequent study demonstrated that the combination of cisplatin and 5FU for radiosensitising chemotherapy was safe, tolerable, and efficacious (Rodel *et al*, 2002b).

A common theme among these studies was the use of salvage radical cystectomy when necessary. Surveillance cystoscopy was performed following combination treatment in the European studies, whereas the RTOG and Massachusetts General Hospital (MGH) studies employed an interval cystoscopy after an ‘induction’ period of treatment to assess the response. Patients with less than complete response to combination therapy were referred for radical cystectomy. The complete response rate found at cystoscopy following induction or completed therapy in these studies was generally in the range of 65–75%. Patients with only carcinoma *in situ*, or with superficial cancer at a new site, were included in the group of complete responders, whereas patients with any muscle-invasive bladder cancer, or with superficial cancer at the original site, were referred for surgery.

In the largest series yet reported, the University of Erlangen recently compiled their data on 415 patients treated with selective bladder preservation over an 18-year period, including the 162 patients described in their prior study (Rodel *et al*, 2002a). Of the 415 patients, 89 had stage T1 disease only, and the remainder had T2–T4. The large majority had no evidence of lymph node metastases. Over 100 patients were treated with radiation alone, whereas 289 were treated with concurrent chemotherapy and radiation. Radiosensitising chemotherapy consisted of cisplatin (145), carboplatin (95), or cisplatin and 5FU (49). Results were reported for all 415 patients as a group, which somewhat obscured the results for patients treated with combination therapy. The complete response rate at interval cystoscopy was significantly higher among patients treated with combination therapy than among patients treated with radiation alone (*P*=0.001). The overall 5-year survival including T1 patients was 51%, and survival with an intact bladder was 42%. The authors also found that initial tumour response was a strong prognostic factor for disease-specific survival and overall survival.

It is important to emphasise the role of transurethral debulking (TURB) in combined modality bladder-preservation treatment. The complete response rate of 65–75% at interval cystoscopy reflects not only the effect of concurrent radiation and chemotherapy, but also the effect of TURB. It is likely that TURB alone would result in a complete response in some patients, and, even in those with persistent microscopic disease after resection, the debulking effect of TURB probably enhances the efficacy of concurrent radiation and chemotherapy. Ability to completely resect visible tumour was a strong predictor of complete response as well as overall survival in univariate and multivariate analyses (Shipley *et al*, 1987; Rodel *et al*, 2002a).

Another critical surgical point is the essential role of salvage cystectomy in incomplete responders to combined modality therapy. Of 190 patients treated with selective bladder preservation, 66 patients (35%) ultimately underwent radical cystectomy for incomplete response to therapy or recurrent invasive tumour (Shipley *et al*, 2002). The 5-year disease-specific survival rate for these patients was 57% for stage T2 and 42% for stage T3–T4a, confirming that salvage surgery is potentially curative therapy for many patients in this program. Importantly, no patients have required cystectomy to treat radiation-induced bladder toxicity.

## RTOG STUDIES

Since the initial publication of RTOG 85-12, this group has completed a series of clinical trials examining combined modality therapy (Table 1

**Table 1:** RTOG bladder-preservation studies

**RTOG number**	**Adjuvant/neoadjuvant therapy**	**Radiation therapy**	**Radiosensitising chemotherapy**	**# of patients**	**5-year survival**	**Reference**
85–12	None	Daily	Cisplatin	42	52%	(Tester *et al*, 1993)
88–02	Neoadjuvant MCV	Daily	Cisplatin	91	62%	(Tester *et al*, 1996)
89–03	+Neoadjuvant MCV	Daily	Cisplatin	123	49%	(Shipley *et al*, 1998)
95–06	None	Hypofractionated	Cisplatin+5FU	34	N/A	(Kaufman *et al*, 2000)
97–06	Adjuvant MCV	Twice daily	Cisplatin	52	N/A	(Hagan *et al*, 2003)
99–06	Adjuvant GC	Twice daily	Cisplatin+paclitaxel	84	N/A	—

MCV – methotrexate, cisplatin, vinblastine; 5FU – 5-fluorouracil; GC – gemcitabine, cisplatin; N/A – not available.

). A major effort was undertaken to determine the role of neoadjuvant chemotherapy in studies 88-02 and, in a randomised phase III trial, in 89-03 (Tester *et al*, 1996; Shipley *et al*, 1998). The majority of patients who relapse following definitive local therapy have distant, rather than local, relapse, indicating the likelihood of microscopic metastatic disease at the time of local therapy. Thus, the use of systemic chemotherapy is employed in an attempt to eradicate such microscopic disease. However, in 89-03, treating patients with two cycles of MCV chemotherapy (methotrexate, cisplatin, vinblastine) prior to combination therapy failed to improve survival or local tumour eradication. At a median follow-up of 5 years, the overall survival was 48% in patients treated with MCV, and 49% in the untreated arm. Cystoscopic complete response was 61% in the MCV arm, and 55% in the control arm. At 5 years, distant metastases were present in 35% of the patients treated with neoadjuvant MCV, and 43% of the untreated patients. None of these differences approached statistical significance. Moreover, because of toxicity, the protocol-completion rate for the MCV arm was only 67%. As a result, some subsequent studies employed adjuvant rather than neoadjuvant chemotherapy. Numerous randomised trials have explored the use of neoadjuvant or adjuvant chemotherapy together with radical cystectomy, a topic beyond the scope of this review (Bajorin, 2001; Raghavan *et al*, 2002; Sternberg, 2002; Grossman *et al*, 2003).

In subsequent RTOG studies, a number of modifications were made to the radiation treatment. High-dose hypofractionation was employed in 95-06, together with both cisplatin and 5FU for radiosensitisation (Kaufman *et al*, 2000). Seven of 34 patients (21%) experienced grade 3 or 4 haematological toxicity, though no deaths resulted. No patients required cystectomy for radiation toxicity, despite the high-dose hypofractionation. Complete response was seen in 67% of patients, and 3-year overall survival was 66%. The RTOG then turned to conventional twice-daily hyperfractionation in 97-06 and subsequent studies. In 97-06, a total of 45.6 Gy was delivered to the pelvis and bladder, and 64.8 Gy to the bladder tumour. Individual fractions were 1.8 Gy to the pelvis with a boost of 1.6 Gy to the bladder tumour during induction treatment, twice a day for 12 days, and then 1.5 Gy fractions to both during consolidation treatment, twice a day for 8 days (Hagan *et al*, 2003). In 99-06, radiation was delivered in a similar fashion with a boost to the whole bladder, and an additional boost to the bladder tumour. The full results of these studies are not yet published.

Initial RTOG studies required hospitalisation of patients to deliver the combined modality treatment. However, since 95-06, all treatments have been administered in the outpatient clinics, with only rare hospitalisations for complications of therapy. This approach has been feasible and safe, and has greatly increased the appeal of combined modality therapy.

## PREDICTORS OF OUTCOME

Initial clinical stage continues to be the most important predictor of overall survival in patients treated for muscle-invasive bladder cancer. In a series of 190 patients treated with selective bladder preservation at the MGH, 5-year overall survival and disease-specific survival were 62 and 63%, respectively, for stage T2, and 47 and 53% for stage T3–T4a (Shipley *et al*, 2002). Similarly, stage was an important predictor of survival with an intact bladder. The 5-year disease-specific survival with an intact bladder was 57% for patients with stage T2, but only 35% for patients with stage T3–T4a. In the series from the University of Erlangen, 5-year overall survival was 75% for stage T1, 56% for T2, 44% for T3, and 17% for T4 (Rodel *et al*, 2002b).

The presence or absence of hydronephrosis at the time of diagnosis has also affected treatment success. Of the 190 patients reviewed at the MGH, 27 had initial hydronephrosis (Shipley *et al*, 2002). Their likelihood of complete response at the first interval cystoscopy was 37%, compared with 68% in patients without hydronephrosis (*P*=0.002). Ultimately, 60% of these patients underwent radical cystectomy, compared with 31% of the other patients (*P*=0.004). Despite the low total numbers of patients with hydronephrosis, this factor approached statistical significance for predicting survival in multivariate analysis. The 5-year overall and disease-specific survivals were 48 and 53% for patients with hydronephrosis, compared with 55 and 64% for patients without hydronephrosis. As a result of this finding, patients with initial hydronephrosis are generally excluded from bladder-preservation protocols.

The initial response to treatment does not predict survival outcome, but is an independent predictor of successful bladder preservation. Not surprisingly, advanced age predicts poorer overall survival for patients treated with selective bladder preservation (Rodel *et al*, 2002b; Shipley *et al*, 2002). However, there is no clear effect on disease-specific survival, suggesting that age may not impact on the success of this treatment approach. Gender has not been found to affect outcome.

## COMPARISON WITH TREATMENT OUTCOMES FROM CONTEMPORARY CYSTECTOMY SERIES

The discrepancy between clinical staging and pathologic staging is an important confounding factor in comparing results of radical cystectomy with results of bladder preservation. Since clinical staging is more likely to understage the extent of disease, there is an outcome bias in favour of surgical series that benefit from pathologic staging. Nevertheless, outcomes from bladder preservation treatment compare favourably to outcomes reported from contemporary surgical series.

Two large, single-institution surgical series have been recently published. The University of Southern California reported on 1054 patients undergoing radical cystectomy (Stein *et al*, 2001). Of these, 633 patients had pathologic stage T2–T4a, and their 5-year overall survival rate was 48%. A second series, from Memorial Sloan Kettering Cancer Center, reported a 5-year overall survival rate of 36% in patients with T2–T4 tumours (Dalbagni *et al*, 2001). Finally, a recent Intergroup study examining neoadjuvant chemotherapy in patients undergoing radical cystecomy for muscle-invasive bladder cancer reported a 5-year overall survival of 50% (Grossman *et al*, 2003). In patients who underwent cystectomy without receiving chemotherapy, the 5-year survival was only 43%. Thus, even contemporary surgical series demonstrate a high rate of disease recurrence for muscle-invasive disease, with cure rates no higher than those that have been observed in current selective bladder-preserving approaches (see Table 1). Comparison is hindered by the lack of a phase III, randomised trial of the two approaches. Unfortunately, cooperation for such an endeavour has not yet been forthcoming from the different involved disciplines.

## QUALITY OF LIFE OUTCOMES

There are legitimate concerns among physicians regarding the long-term effects of high-dose bladder radiation on bladder function, as well as the potential harmful effects to the bowel and to erectile function in men. In earlier cross-sectional studies from Italy and Sweden, over 74% of patients reported good long-term urinary function, and low levels of patient-reported systems (Caffo *et al*, 1996; Henningsohn *et al*, 2002). A recent study from the MGH has studied the quality of life and urodynamics in a comprehensive fashion among 49 patients who had completed bladder preservation therapy a median of 6.3 years earlier (Zietman *et al*, 2003). In all, 32 patients agreed to undergo formal urodynamic evaluation. Of these, 24 were judged to have bladders with normal function. The median volume voided was 284 cm^3^ (range 125–630 cm^3^), and median bladder capacity was 400 cm^3^ (range 221–747). Five of the 18 men studied had evidence of reduced flow. Three had post-void residual of greater than 100 cm^3^, and two of these had urodynamic evidence of bladder outlet obstruction. Thus, impaired urinary function may have been due to external factors and not to impaired bladder function *per se*.

One-fifth of all patients in the study reported some degree of incontinence within the preceding week, and women were twice as likely as men to report incontinence. Mild to moderate bowel symptoms were reported by 20% of men and 27% of women. The symptom most frequently reported was rectal urgency. A total of 14% reported moderate or greater symptoms, but no patients reported severe or very frequent bowel symptoms. In two prior studies, 10 and 32% of patients complained of moderate or greater bowel symptoms. Sexual function among men was remarkably intact, with 8% reporting dissatisfaction with their sex life, 33% reporting neither satisfaction nor dissatisfaction, and 59% reporting satisfaction (Zietman *et al*, 2003). Sildenafil was used by 16% of men, perhaps accounting in part for the favourable numbers compared to earlier studies, in which only 25–40% of men retained adequate sexual function (Caffo *et al*, 1996; Henningsohn *et al*, 2002). The numbers in this study compare favourably to patients who undergo radiation for prostate cancer. No data were available for sexual function among women in this study.

The major conclusions of this recent study from the MGH were that normal bladder function was preserved in the majority of patients with an intact bladder years after bladder preservation therapy, that about one-fifth of patients complained of mild to moderate bowel symptoms, and that one-fifth of patients suffered from occasional urinary incontinence.

## CONCLUSIONS

The use of bladder-preservation therapy for muscle-invasive TCC of the bladder is a valid alternative to radical cystectomy in selected cases. Long-term efficacy is comparable to radical cystectomy, with the advantage of preserving excellent bladder function in the majority of long-term survivors. Contemporary protocols utilise a combination of transurethral tumour resection, concurrent radiation and radiosensitising chemotherapy, and often adjuvant chemotherapy. These approaches require close coordination among surgical, radiation, and medical oncologists. Treatments are well tolerated, and are carried out in the outpatient setting. Future investigation will optimise the use of systemic therapy during selective bladder preservation, and will explore the role for rationally targeted biologic agents in the management of locally advanced bladder cancer.
